# Cryo-EM reveals an entangled kinetic trap in the folding of a catalytic RNA

**DOI:** 10.1126/sciadv.abq4144

**Published:** 2022-08-26

**Authors:** Steve L. Bonilla, Quentin Vicens, Jeffrey S. Kieft

**Affiliations:** ^1^ Department of Biochemistry and Molecular Genetics, University of Colorado Anschutz Medical Campus, School of Medicine, Aurora, CO 80045, USA.; ^2^ RNA BioScience Initiative, University of Colorado Anschutz Medical Campus, School of Medicine, Aurora, CO 80045, USA.

## Abstract

Functional RNAs fold through complex pathways that can contain misfolded “kinetic traps.” A complete model of RNA folding requires understanding the formation of these misfolded states, but they are difficult to characterize because of their transient and potentially conformationally dynamic nature. We used cryo–electron microscopy (cryo-EM) to visualize a long-lived misfolded state in the folding pathway of the *Tetrahymena thermophila* group I intron, a paradigmatic RNA structure-function model system. The structure revealed how this state forms native-like secondary structure and tertiary contacts but contains two incorrectly crossed strands, consistent with a previous model. This incorrect topology mispositions a critical catalytic domain and cannot be resolved locally as extensive refolding is required. This work provides a structural framework for interpreting decades of biochemical and functional studies and demonstrates the power of cryo-EM for the exploration of RNA folding pathways.

## Teaser

Cryo-EM reveals the structure of a catalytic RNA in a previously mysterious misfolded state.

## INTRODUCTION

To function, RNAs must find their native three-dimensional (3D) fold among a multitude of alternative conformations in a biologically relevant time scale (*1*, *2*). This process is not trivial; the high stability and promiscuity of base-base interactions create an intrinsic thermodynamic propensity to form non-native contacts, potentially trapping the RNA in stable misfolded states and slowing down the folding process (*1*–*4*). So-called “kinetic traps” have been detected in the folding pathways of several model RNAs and are more likely in large RNAs with intricate 3D folds (*5*–*7*). In vivo, kinetic traps may be resolved by the action of chaperones that actively misfold the RNA and allow it to refold and/or by the binding of proteins that bias the folding pathway toward the functional fold (*8*–*10*). Understanding the principles governing formation and resolution of misfolded states is thus a critical step toward a complete model of RNA folding. However, direct visualization of these states has not been generally possible by crystallography because they may be transient and/or conformationally dynamic. Dynamic states may be observed using nuclear magnetic resonance, but detailed structural information is limited by the size of the RNA. Given the latest advances in the ability of cryo–electron microscopy (cryo-EM) to solve dynamic RNA-only structures (*11*–*14*), we reasoned that this technique may offer a way to directly observe the 3D structure of RNA folding intermediates, including misfolded states, which have been elusive to structural biology.

**Fig. 1.**
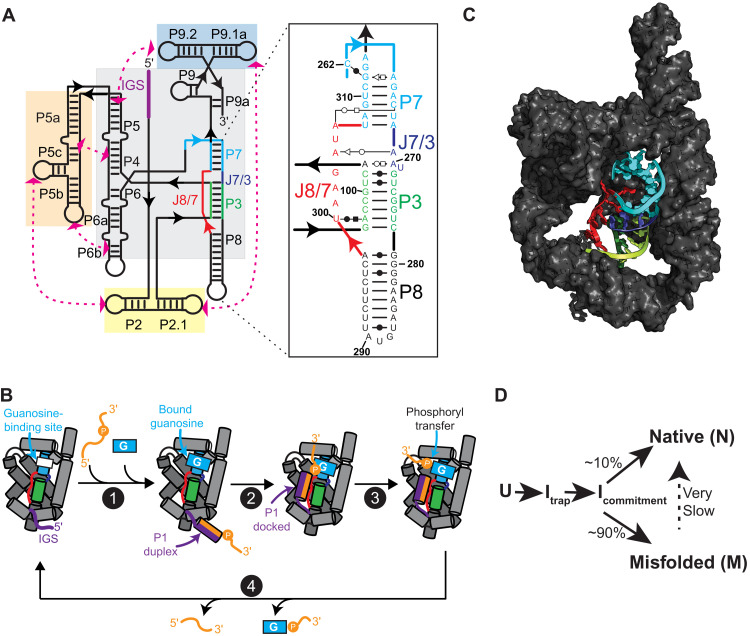
Structure and function of TET ribozyme. (**A**) Schematic of the secondary structure TET. Paired regions (P) and junctions (J) are named using conventional nomenclature. Gray and colored boxes highlight core and peripheral domains, respectively. IGS, internal guide sequence. Inset shows secondary structure of domains in the proximity of P7. (**B**) Simplified catalytic cycle of multiturnover TET ribozyme. For brevity, multiple processes are illustrated as single steps. For a detailed mechanistic description, see (*15*). (1) Oligo substrate anneals to internal guide sequence to form P1 and exogenous guanosine binds to pocket in P7. (2) P1 docks into the core. (3) Phosphoryl transfer between guanosine nucleophile and oligo substrate and undocking of P1. (4) Release of products to regenerate *apo* ribozyme. Colors as in (A). (**C**) Published cryo-EM structure of *apo* L-21 Sca I TET ribozyme in its native folded state [Protein Data Bank (PDB) ID, 7ez0]. P7, P3, P8, J8/7, and J7/3 are shown in cartoon representation and colored as in (A). (**D**) Simplified folding pathway of TET ribozyme at 25°C in the presence of 10 mM Mg^2+^. U is the unfolded state. I_trap_ is hypothesized to be a largely structured misfolded intermediate (*22*). I_commitment_ is a common intermediate that precedes commitment to pathways leading to N or M (*22*).

The self-splicing *Tetrahymena thermophila* group I intron, the first catalytic RNA found, and its multiturnover ribozyme derivative (herein referred to as TET; Fig. 1A) are well-established model systems used for decades to dissect general principles of RNA folding and catalysis (*15*–*18*). TET catalyzes the cleavage of a substrate strand using an exogenous guanosine nucleophile (Fig. 1B). This ~125-kDa ribozyme folds into a compact structure with an internal core of stacked helices stabilized by tertiary contacts between surrounding peripheral domains (Fig. 1C).

Several intermediates have been identified in the folding pathway of TET, including a long-lived misfolded state (referred to as “M”), which can be experimentally accumulated (*19*–*23*). At standard in vitro conditions, ~10% of molecules fold directly to the native state (N) and ~90% to M (Fig. 1D). Refolding from M to N is very slow, in the time scale of hours, suggesting that this process requires considerable structural reorganization (*22*, *23*). Consistent with this, solution conditions and mutations that destabilize RNA tertiary structure accelerate refolding from M to N (*23*). Mutagenesis of the ribozyme core demonstrated that formation of non-native base pairs leads to M; thus, it was initially hypothesized that M contains non-native secondary structure elements near the catalytic center (*22*, *24*, *25*). Paradoxically, hydroxyl radical and dimethyl sulfate footprints of M and N show only minor differences localized mostly to the functionally important P7 helix (Fig. 1, A and C), suggesting that M and N essentially form the same secondary structure and tertiary contacts (*23*).

To explain the paradoxical nature of M, it was hypothesized that, although M and N form nearly identical structures, two single-stranded elements are crossed incorrectly in M, resulting in a non-native trapped topology that requires extensive unfolding to be resolved (*23*). According to this model, the alternative secondary structure supported by mutations biases the folding pathway toward M and traps the ribozyme in the incorrect topology, but it is later replaced by native-like contacts before forming M. Although this hypothesis is consistent with the biochemical and functional data, the 3D structure of the M state has remained elusive, and the topological isomer model has remained to be tested directly.

Recently, complete structures of *apo* and *holo* TET ribozymes in the N state were solved by cryo-EM (*12*, *14*). Concurrent with those studies, we applied cryo-EM to solve the structure of both the N and the M states of TET. By directly observing and comparing these two states, we rationalize decades of studies and paradoxical observations and demonstrate the power of cryo-EM to dissect RNA folding intermediates.

## RESULTS

### Cryo-EM reveals two major conformational states of TET

**Fig. 2.**
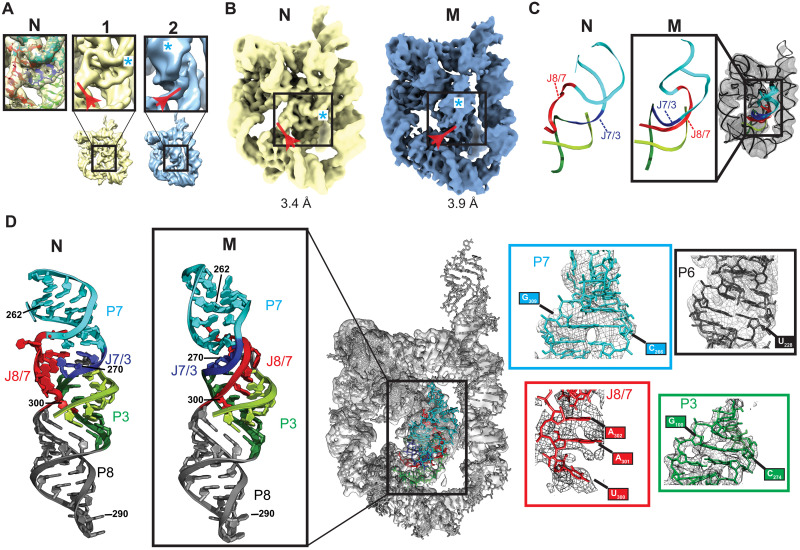
Cryo-EM studies of TET folded at room temperature revealed two distinct conformational states. (**A**) Particle classification revealed two distinct conformational classes (naps 1 and 2; figs. S1 and S2). Major differences within the core are shown in boxes. Density corresponding to J8/7 is marked with a red arrow. Density corresponding to the minor groove of P7 is marked with a cyan asterisk. Left box shows published structure of N fitted to map 1. (**B**) Maps of N (yellow) and M (blue) states, corresponding to refined versions of maps 1 and 2 in (A), respectively. (**C**) Models generated by autoDRRAFTER superimposed on cryo-EM map. Representative modeled structure is enlarged and compared to the same region of N. (**D**) Atomic model of the full M state after refinements. Refined structure is docked into cryo-EM map (middle). Atomic model of the P7-P3-P8 stack in M is compared to that of N (far left) to highlight structural differences between these two states. Close-ups show examples of structural models fitted to density (right).

Previous studies showed that M can be enriched (~90%) by folding TET at 25°C in the presence of 10 mM Mg^2+^ (*23*). We therefore in vitro transcribed and purified the L-21 Sca I ribozyme sequence and folded the RNA using those conditions and then immediately prepared cryo-EM samples and imaged using a 300-kV Krios cryo-EM microscope (fig. S1 and table S1). Ab initio 3D reconstructions and refinements using the cryoSPARC software (*26*) produced maps consistent with the global structure of TET, but the density at the core was not well defined and suggested a mixture of states (fig. S1). Similarly, unsupervised 3D classification using the Relion software (*27*) revealed heterogeneity localized to the ribozyme core, but the resulting maps did not display sufficient resolution to unambiguously differentiate the core conformations (fig. S2). To address this, we first performed 3D variability analysis, which uses probabilistic principal components analysis to fit a linear subspace describing variability in the particles (*28*), to generate multiple maps across the first principal component (movie S1 and fig. S3). These maps were then used as references for particle 3D classification and refined in cryoSPARC (fig. S3). Comparison of the refined maps revealed two conformational classes, with major differences localized to the core of the ribozyme (Fig. 2A and fig. S3). Other volumes generated by particle classification could be assigned to one of the two conformational classes (fig. S3). The best quality maps from each class were refined to 3.4- and 3.9-Å resolution (Fig. 2B and fig. S4).

The first class (Fig. 2, A and B, yellow), which was refined to 3.4 Å, matches a published cryo-EM map of TET in the N state [map-in-map correlation coefficient (CC), 0.94], and the published structure docks well into the map without additional refinements (average CC_mask_, 0.77; fig. S5). The map model correlation per residue was essentially the same with the published map and with our map (fig. S5). Therefore, this class represents a population of N in the sample, consistent with previous studies showing that a fraction of molecules fold directly to N under the experimental conditions (Fig. 1D) (*22*, *23*). In contrast, the second class (Fig. 2, A and B, blue), which was refined to 3.9 Å, does not fit the structure of N and revealed major differences in the core near the functionally important P7 helix, which contains the conserved guanosine-binding site, indicating that this class likely corresponds to the M state.

As expected, most (~74%) particles were classified into the M state (fig. S3B). However, the proportion of particles classified into M versus N does not match the previously determined partitioning of 90% into M and 10% into N (*23*). This is likely due to current limitations related to cryo-EM data acquisition and analysis that prevent particle classification from being quantitative. These limitations include the low signal-to-noise ratio of individual particle images and imperfect particle picking and junk removal.

### 3D structures reveal topological differences between the N and M states

The core of TET contains two sets of stacked helices: P4-P5-P6 and P3-P7-P8-P9 (Fig. 1A. gray box). J8/7 is an unpaired stretch of seven nucleotides that links P7 to P8 and makes contacts with the P3 helix (Fig. 1A). Comparison of N and M maps suggested that the peripheral domains and the P4-P5-P6 stack of the core were similar between the two conformations, although with minor differences discussed below. In contrast, the maps suggested that J8/7 extends in a different direction in each map (Fig. 2, A and B, red arrow) and that P7 is rotated in M relative to its position in N (Fig. 2, A and B, cyan asterisk). To learn more about these conformational differences, we fitted an atomic model into the density of M. As the resolution was not sufficiently high for manual fitting, we used autoDRRAFTER (*13*) for map-guided computational modeling of the core of M, keeping the peripheral elements, the P4-P5-P6 stack, and the secondary structure fixed during modeling (Fig. 2C and fig. S6). The full structure of M was refined using Phenix and Coot modeling software (Fig. 2D and fig. S6) (*29*, *30*). The final structure fits well into the map (CC_mask_, 0.74) without major steric clashes (clash score, 6.61). While the moderate resolution of the map in the core does not allow atomic-level precision, the strand topology and global structure of M and its comparison to the known structure of N are clear and provide important insights.

The final model of M reveals a core topology that diverges from that of N (Fig. 2, C and D). Whereas in N, the single strand J8/7 is “on top” of J7/3 (which joins helices P7 and P3) as seen from the orientation shown in Fig. 2C, in M J8/7 passes “under” J7/3. Further, the position of J8/7 in M strongly suggests that it docks into the major groove of P3, largely differing from its position away from the major groove of P3 in N; however, the resolution of the structure of M in this area is not sufficiently high to unambiguously describe the specific interactions made between J8/7 and P3. The entanglement of J8/7 is accompanied by a ~90° rotation of P7, likely induced by the new position of J8/7 that constrains proper placement of P7 (Fig. 2D). In contrast, helix P3 is essentially unmoved relative to its native position (Fig. 2D). Consistent with previous observations, long-range tertiary contacts are maintained, although large rearrangements occur within the core. These observations are consistent with the topology isomer hypothesis discussed above (*23*, *31*), although the specific topological differences and the identities of the entangled strands differed from those predicted. Visual inspection shows that this topological error cannot be resolved locally and requires major unfolding of the ribozyme (vide infra), consistent with the hours-long time scale at which the M to N transition occurs.

**Fig. 3.**
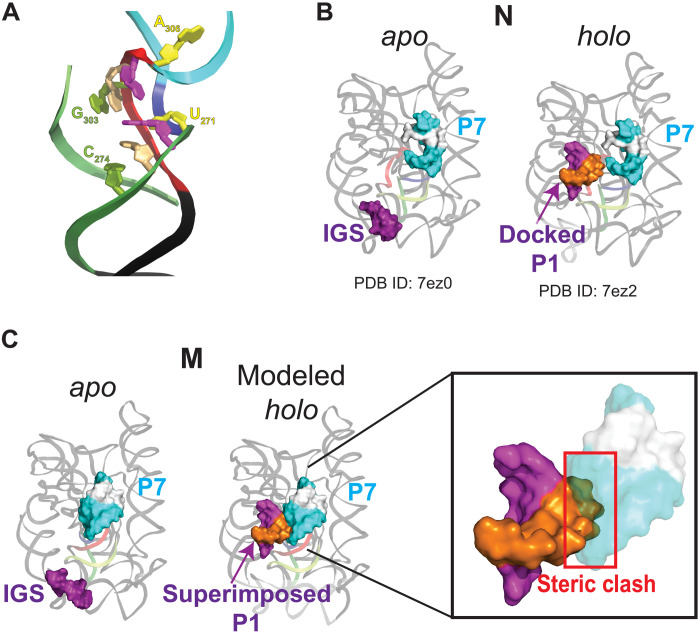
Structure of M provides insights into ribozyme misfolding and catalysis. (**A**) Nucleotides predicted to base pair in alt-P3, leading to the formation of M. Backbone is colored as in Fig. 2. Bases predicted to pair in alt-P3 are the same color. (**B**) Published cryo-EM structures of *apo* L-21 Sca I (PDB ID, 7ez0) and *holo* L-16 Sca I (PDB ID, 7ez2) TET ribozymes folded in the native functional state. For clarity, helix P10 and additional base pairs formed by a second oligo substrate in the published structure (*12*) are not shown. P7 and internal guide sequence are rendered as surfaces and nucleotides in P7 that form the binding pocket for guanosine are colored white. (**C**) Superposition of docked P1 (extracted from PDB ID 7ez2) on structure of M state. Inset shows steric clash predicted to block docking of P1 into the core of the M state.

The structure of M is consistent with previous studies showing a strong link between the formation of an alternative P3 (alt-P3) secondary structure and formation of M (*24*, *25*). In alt-P3, nucleotides 303 to 306 in J8/7 are proposed to base pair with nucleotides 271 to 274, effectively preventing formation of the long-range native P3 (Fig. 1A). On the basis of the nearly identical chemical probing footprints of M and N, it was later proposed that formation of alt-P3 traps the ribozyme in the incorrect topology, but alt-P3 is later replaced by the native P3 (*23*). The map of M, although not allowing unambiguous positioning of every nucleotide, supports this model. In M, J8/7 docks into the major groove of P3 and is near nucleotides 271 to 274, where it could readily pair to form alt-P3 (Fig. 3A). Thus, only local rearrangements are required to transition from an intermediate state with alt-P3 to the M state. Although we cannot make any conclusions about the conformation of these elements before M is formed, the structure suggests how formation of alt-P3 could position J8/7 in its final trapped position in M.

### The M-state structure explains its decreased catalytic activity

Given the similarity of the structures of N and M suggested by chemical probing, the difference in their catalytic activity was puzzling, but the 3D structure of M now resolves this paradox. As shown in a simplified scheme of the TET catalytic cycle (Fig. 1B), the P1 duplex—formed by base pairing of the substrate strand to the internal guide sequence—docks into the core, positioning the 5′ splice site in proximity to the bound guanosine nucleophile. Structures of N with and without bound substrates showed that the core is globally preorganized for P1 docking (Fig. 3B) (*12*). This is supported by solution small-angle x-ray scattering (SAXS) data, which are consistent with no major changes in the overall structure of the ribozyme upon substrate binding (*32*).

In M, the P1 docking site is partially occupied by P7, which is rotated ~90° and moved by several angstroms relative to N (Fig. 3C). Thus, the rotated P7 effectively blocks P1 docking, which is an essential step early in the catalytic cycle. Although particle variability analysis (movie S1) and the lower local resolution of P7 in M (fig. S7) suggest that P7 is conformationally dynamic, this helix is unable to rotate to its native position without major rearrangements due to constraints imposed by the connecting strands. It was previously hypothesized that the inactivity of M was caused by rearrangements preventing P1 docking on the basis of a cleavage assay of a 3′ splice site mimic oligo that does not require P1 docking (*23*). The 3D structure of M strongly supports this hypothesis. Moreover, in the context of the complete group I intron and on the basis of a structure of TET mimicking the second step of splicing (*12*), the position of P7 would also interfere with the formation of P10 before the second transesterification reaction, making M incompetent for self-splicing. Last, as the guanosine-binding site is contained within P7, the large rotation and displacement of this helix would cause the bound guanosine to be in a different position relative to N, even if P1 was able to dock. Overall, the alternative position of P7, which is coupled to the entangled J8/7, explains the functional differences between N and M.

### M contains small differences in the relative positions of the peripheral domains

Although large differences between M and N are observed at the core, their peripheral domains are very similar [root mean square deviation (RMSD)_backbone_, 2.43 Å; fig. S8]. M forms native long-range tertiary contacts consistent with previous functional studies of ribozyme mutants (*23*). However, there are small differences in the relative position of the peripheral domains, presumably arising from the distinct core conformations. To better visualize these differences, we superimposed the P4-P6 domains (nucleotides 107 to 258) of N and M, which are nearly identical in the two states (RMSD, 0.995 Å; fig. S8). With the P4-P6 domain superimposed, the backbone of other peripheral domains is misaligned by up to ~9 Å, most noticeably in P2 and P9 (fig. S8). Previous SAXS studies suggested a 10% increase in the radius of gyration (*R*_g_) of M versus N (*32*). We do not observe significant differences in compaction, and both structures have essentially the same *R*_g_ of ~38 Å. However, the local resolution distributions are very different between N and M, suggesting differences in global flexibility (fig. S7). In particular, P9, which is conformationally dynamic (*12*), appears to be much more flexible in M relative to N, as inferred by the local resolution of this domain (fig. S7). Thus, differences in *R*_g_ observed by SAXS may be reflective of differences in the direction and/or magnitude of these dynamics, the presence of other less-compact conformational species in the SAXS sample, or of limitations in the accuracy of *R*_g_ estimates from SAXS profiles.

### A model for disentangling the topological error

Resolution of the topological error in M cannot occur with only local rearrangements, raising the question of how much unfolding is needed to allow the transition from M to N. Although our data cannot establish the mechanism of this transition, comparing the structures of M and N in the context of previous functional and biochemical studies can provide insights. We generated a model for the transition of M to N that requires minimal secondary structure disruption and that is consistent with previous studies (movie S2). In this model, tertiary contacts between peripheral domains and the long-range base pairs that form P3 break to allow rotation of a long hairpin that includes P7, J8/7, J7/3, nucleotides 272 to 278 of P3, and P8 (movie S2). P7 rotates ~90° to reach its native position, while P8 undergoes an ~360° rotation that is allowed by the flexibility of single strands J8/7, J7/3, and nucleotides 272 to 278. After these rotations, refolding P3 and docking of the tertiary contacts results in formation of N (movie S2). This model shows that there are paths from M to N that do not require extensive breaking of secondary structure. This model is also in agreement with observations that destabilizing tertiary interactions between peripheral domains and/or base pairs in P3 increases the rate constant for the transition from M to N (*23*, *31*). Further, because minimal disruption of secondary structure is required, the model is consistent with the ability of M to convert to N at physiological temperatures, albeit slowly.

## DISCUSSION

RNA folding pathways are complex, with multiple branching points and intermediates. For decades, researchers have applied a wide array of diverse tools to detect folding intermediates and misfolded states in the folding of model RNAs. These techniques include quantitative thermodynamic and kinetic analysis, single-molecule imaging, x-ray scattering techniques, and electrophoretic mobility assays, among others (*33*). Pathways deduced by these techniques have proven invaluable to our understanding of RNA folding and can generate quantitative, testable predictions. What has been lacking is a 3D view of the conformational species populating the pathways. This information would provide a structural framework for interpreting decades of functional and biochemical studies and generate additional hypotheses, ultimately enhancing our predictive understanding of the folding process. The scarcity of 3D structural information is in part due to a lack of tools to readily explore the structures of these perhaps transient and/or conformationally dynamic states. Building on previous functional and biochemical studies and taking advantage of the recent advances in cryo-EM, we used the *T. thermophila* group I intron ribozyme as a model for RNA misfolding and solved the structure of a long-lived misfolded intermediate state that had remained mysterious for decades. In so doing, we demonstrate the power of cryo-EM as a tool to explore dynamic folding pathways of complex, functional RNAs.

The term “misfolded RNA” might suggest the formation of non-native secondary structures or global changes in tertiary structure. While these states can form, in the case of the long-lived misfolded state of the *T. thermophila* group I intron, referred to as the “M” state, misfolding is generated by a pair of incorrectly crossed unpaired strands that cause a topological error within a fold with native-like secondary structure and tertiary contacts. This topological error results in the large rotation of a functionally important domain, effectively preventing organization of the catalytic site and rendering the M state functionally incompetent. The question of how often topological errors arise within the folding pathways of other complex RNA folds is not clear, but the structure of the M-state model provides an example that will facilitate future explorations.

Given the inherent thermodynamic propensity of RNAs to misfold, it is remarkable that biology has found ways to produce functional RNAs in time scales that are consistent with life. In part, kinetic traps may be alleviated in vivo by the work of chaperones with helicase activity and/or the stepwise binding of proteins that guide the folding process. In addition, cotranscriptional folding may bias the folding pathway. A mechanistic understanding of these complex processes requires the understanding of the inherent folding properties of RNA in isolation. Experimental and computational advances have turned cryo-EM into a premier tool to solve the high-resolution native structures of biological macromolecules. Ongoing developments in time-resolved cryo-EM promise dissection of transient intermediate structures (*34*). Here, we demonstrate the application of conventional cryo-EM to understand the structure of a transient but long-lived misfolded intermediate in a complex, folded RNA. This state was not directly observed for many years, in part because of limitations of other structural methods. Now, direct observation of the state provides strong evidence in support of previous models proposed on the basis of functional and biochemical data (*23*, *31*). The remarkable fact that these models were largely correct, even in the absence of a 3D structure, illustrates the power of rigorous quantitative biochemistry and biophysics to sort out complex problems in RNA structure and folding and the natural marriage between these methods and cryo-EM.

## MATERIALS AND METHODS

### Preparation of folded TET RNA

We ordered a DNA fragment (gBlocks, Integrated DNA Technologies) containing the L-21 Sca I TET sequence flanked by the T7 RNA polymerase promoter sequence. The DNA fragment was polymerase chain reaction (PCR)–amplified, purified using the GeneJET PCR Amplification Kit (Thermo Fisher Scientific), and used as a template for in vitro transcription.

TET RNA was transcribed in a 250-μl reaction containing 6 mM of each nucleotide triphosphate (adenosine 5′-triphosphatase, cytidine 5′-triphosphate, guanosine 5′-triphosphate, and uridine 5′-triphosphate), 60 mM MgCl_2_, 30 mM tris (pH 8.0), 10 mM dithiothreitol, 0.1% spermidine, 0.1% Triton X-100, RNasin ribonuclease (RNase) inhibitor (0.24 U/μl; Promega), T7 RNA polymerase (~0.14 mg/ml; in-house prepared), and 37 μM DNA template. The reaction was incubated at 37°C for 3 hours and ethanol-precipitated overnight. RNA was purified by denaturing gel electrophoresis (5% polyacrylamide) and by AMPure XP magnetic beads (Beckman Coulter). The purified RNA was buffer-exchanged three times into RNase-free water using 30-kDa cutoff Amicon centrifugal filters (Millipore). The RNA was stored at −20°C until preparation of cryo-EM grids.

Before preparation of cryo-EM grids, the RNA was folded in 10 mM MgCl_2_ and 50 mM Na-Mops (~30 mM Na^+^) (pH 7.0) by incubating at 25°C for 15 min. These conditions accumulate the misfolded state of the ribozyme (*23*).

### Cryo-EM grid preparation

A Gatan Solarus model 950 advanced plasma system was used to clean C-Flat holey carbon grids (hole size, 1.2 μm; spacing, 1.3 μm; mesh, 400; Electron Microscopy Sciences). Settings used for the plasma cleaning were “Cleaning time,” 6 s; “Vacuum target,” 70 mtorr; “Vacuum range,” 0 mtorr; “Pumping switch point,” 20 torr; “Turbo pump speed,” 750 Hz; “O_2_ gas flow,” 27.5 standard cubic centimeters per minute (SCCM); “H_2_ gas flow,” 6.4 SCCM; “Air gas flow,” 0.0 SCCM; “Gas flow timeout,” 20 s; “Forward RF (radio frequency) target,” 50 W; “Forward RF range,” 5 W; “Maximum reflected RF,” 5 W; “RF tuning timeout,” 4 s; “RF tuning attempts,” 3. Then, 3 μl of folded RNA solution [final concentrations: 16.8 μM RNA, 10 mM MgCl_2_, and 50 mM Na-Mops (~30 mM Na^+^) (pH 7.0)] was deposited on each grid, and an FEI Vitrobot Mark IV was used to plunge-freeze the grids using liquid ethane. The humidity of the Vitrobot chamber was set to 100% and the temperature to 4°C; blot force was set to −5; blot time was 2.5 s with 0-s wait time. Filter paper was used for blotting (prod no. 47000-100, Ted Pella Inc.).

### Data collection and analysis

Data were collected at the Pacific Northwest Center for Cryo-EM (PNCC) with a 300-kV Thermo Fisher Scientific Krios transmission electron microscopy in super resolution mode (pixel size, 0.5395 Å), equipped with a Falcon 3 direct electron detector and a Bioquantum K3 imaging filter, and using SerialEM data collection software. We collected 6222 movies (46 frames) with a total dose of 32 *e*/Å^2^ and a defocus range of 0.8 to 2.0 μm (table S1).

Data were processed using cryoSPARC (figs. S1 and S2) (*26*). Imported movies were subjected to patch motion correction and CTF (contrast transfer function) estimation with default parameters (fig. S1). Micrographs were curated to eliminate those with damaged areas, excessive ice contamination, and/or poor CTF estimation, resulting in 5709 curated micrographs. Automated particle picking and extraction (extraction box size, 480 pixels; Fourier crop to box size, 256 pixels) resulted in 4,272,705 putative particles. To remove junk, three rounds of 2D classification were performed (number of classes, 200; circular mask diameter, 170 Å; final full iterations, 2; online-EM iterations, 40; batch size per class, 500). In the last two rounds, the initial classification uncertainty factor was set to 10 to obtain a higher diversity of good classes. A total of 803,573 particles remained after 2D classification.

The particles were used to build three ab initio models. Two of the models (88% of particles; fig. S1) displayed RNA features and global structures consistent with TET. The third map (12% of the particles; fig. S1) was used as a “sink” for removal of junk and suboptimal particles in three rounds of 3D classification (heterogeneous refinement), as done previously (*11*). A total of 650,948 particles remained after 3D classification (*n*_1_ = 285,646; *n*_2_ = 365,302; fig. S2A). The two TET maps were refined using “homogeneous refinement” with default parameters. To resolve remaining conformational heterogeneity, the refined particles were subjected to 3D particle variability analysis (number of modes to solve, 2; filter resolution, 6 Å) (*28*) to generate four “frames” along the first principal component for each of the two original maps (fig. S2A). Total particles (*n* = 650,948) were reclassified between the eight frames and refined (volumes 1 to 8; fig. S2A). The maps were inspected and classified into two major classes based on differences in core density (fig. S2B). Maps with the highest resolutions (volumes 5 and 7; fig. S2A) were subjected to an additional (nonuniform) refinement. The final maps of N and M were refined to 3.4 Å (*n*_N_ = 92,828 particles) and 3.9 Å (*n*_M_ = 98,071 particles), respectively.

### Structural modeling of M state

A schematic of the methods used for structural modeling is provided in fig. S5. The structure of the L-21 Sca I TET ribozyme [Protein Data Bank (PDB) ID, 7ez0], with Mg^2+^ ions removed, was docked into the 3.9-Å resolution map of the M state, using UCSF Chimera (fig. S5A) (*35*). The docked structure was then imported into Phenix (*29*) and a real-space refinement was performed (macro cycles, 1), constraining the secondary structure. Nucleotides corresponding to P7, P3, J8/7, J7/3, and connecting junctions were removed from the structure and were modeled using Rosetta RNA fragment assembly implemented in autoDRRAFTER (fig. S5B) (*13*). The truncated structure (containing the peripheral domains and P4-P6, which fit well into the map) was kept constant during modeling and was provided as an input to autoDRRAFTER, along with the sequence and secondary structure of TET and the 3.9-Å map. The protocol described under “Manually setting up an autoDRRAFTER run” in the ROSIE web server was followed (*36*). Three rounds of autoDRRAFTER modeling were performed (cycles, 30,000; number of models, 2000). The modeled structures converged toward a common topology (fig. S5A). The top 10 models were inspected in the context of the map; all 10 models fit equally well, and one of them was chosen for further refinements. The model was imported into Phenix for a real-space refinement (macro cycles, 5) with secondary structure constraints. Coot (*30*) was used to make minor adjustments to the structure around J8/7, and a final real-space refinement was performed in Phenix (macro cycles, 5). Structures were visualized using PyMOL (Schrödinger Inc.) and UCSF ChimeraX (*37*). *R*_g_ was calculated using CRYSOL (*38*). To generate the model for transition from M to N (movie S2), we manually modified structures using PyMOL to generate intermediate structures. The geometries of the intermediate structures were regularized using Phenix geometry minimization tools and molecular dynamics flexible fitting in Namdinator (*39*), using synthetic electron density maps generated in Chimera as inputs. The intermediate structures were interpolated using “morph conformations” (interpolation method: corkscrew; interpolation rate linear) in Chimera to generate movie S2.

## Supplementary Materials

### This PDF file includes:

Figs. S1 to S8 Table S1 References

## Notes

View/request a protocol for this paper from *Bio-protocol*.

## References

[1] D. Herschlag, RNA chaperones and the RNA folding problem. J. Biol. Chem. 270, 20871–20874 (1995). 7545662 10.1074/jbc.270.36.20871

[2] D. Thirumalai, N. Lee, S. A. Woodson, D. Klimov, Early events in RNA folding. Annu. Rev. Phys. Chem. 52, 751–762 (2001). 11326079 10.1146/annurev.physchem.52.1.751

[3] D. K. Treiber, J. R. Williamson, Exposing the kinetic traps in RNA folding. Curr. Opin. Struct. Biol. 9, 339–345 (1999). 10361090 10.1016/S0959-440X(99)80045-1

[4] S. A. Woodson, Recent insights on RNA folding mechanisms from catalytic RNA. Cell. Mol. Life Sci. 57, 796–808 (2000). 10892344 10.1007/s000180050042 PMC11147119

[5] S. A. Walstrum, O. C. Uhlenbeck, The self-splicing RNA of *Tetrahymena* is trapped in a less active conformation by gel purification. Biochemistry 29, 10573–10576 (1990). 2271667 10.1021/bi00498a022

[6] T. Pan, T. R. Sosnick, Intermediates and kinetic traps in the folding of a large ribozyme revealed by circular dichroism and UV absorbance spectroscopies and catalytic activity. Nat. Struct. Biol. 4, 931–938 (1997). 9360610 10.1038/nsb1197-931

[7] D. M. Chadalavada, S. M. Knudsen, S. Nakano, P. C. Bevilacqua, A role for upstream RNA structure in facilitating the catalytic fold of the genomic hepatitis delta virus ribozyme. J. Mol. Biol. 301, 349–367 (2000). 10926514 10.1006/jmbi.2000.3953

[8] R. Russell, RNA misfolding and the action of chaperones. Front. Biosci. 13, 1–20 (2008). 17981525 10.2741/2557 PMC2610265

[9] S. A. Woodson, S. Panja, A. Santiago-Frangos, Proteins that chaperone RNA regulation. Microbiol. Spectr. 6, 10.1128/microbiolspec.RWR-0026-2018, (2018). 10.1128/microbiolspec.rwr-0026-2018 PMC6086601 30051798

[10] M. L. Rodgers, S. A. Woodson, A roadmap for rRNA folding and assembly during transcription. Trends Biochem. Sci. 46, 889–901 (2021). 34176739 10.1016/j.tibs.2021.05.009 PMC8526401

[11] S. L. Bonilla, M. E. Sherlock, A. MacFadden, J. S. Kieft, A viral RNA hijacks host machinery using dynamic conformational changes of a tRNA-like structure. Science 374, 955–960 (2021). 34793227 10.1126/science.abe8526 PMC9033304

[12] Z. Su, K. Zhang, K. Kappel, S. Li, M. Z. Palo, G. D. Pintilie, R. Rangan, B. Luo, Y. Wei, R. Das, W. Chiu, Cryo-EM structures of full-length *Tetrahymena* ribozyme at 3.1 Å resolution. Nature 596, 603–607 (2021). 34381213 10.1038/s41586-021-03803-w PMC8405103

[13] K. Kappel, K. Zhang, Z. Su, A. M. Watkins, W. Kladwang, S. Li, G. Pintilie, V. V. Topkar, R. Rangan, I. N. Zheludev, J. D. Yesselman, W. Chiu, R. Das, Accelerated cryo-EM-guided determination of three-dimensional RNA-only structures. Nat. Methods 17, 699–707 (2020). 32616928 10.1038/s41592-020-0878-9 PMC7386730

[14] D. Liu, F. A. Thélot, J. A. Piccirilli, M. Liao, P. Yin, Sub-3-Å cryo-EM structure of RNA enabled by engineered homomeric self-assembly. Nat. Methods 19, 576–585 (2022). 35501384 10.1038/s41592-022-01455-w

[15] K. Kruger, P. J. Grabowski, A. J. Zaug, J. Sands, D. E. Gottschling, T. R. Cech, Self-splicing RNA: Autoexcision and autocyclization of the ribosomal RNA intervening sequence of *Tetrahymena*. Cell 31, 147–157 (1982). 6297745 10.1016/0092-8674(82)90414-7

[16] T. R. Cech, Ribozymes, the first 20 years. Biochem. Soc. Trans. 30, 1162–1166 (2002). 12440996 10.1042/bst0301162

[17] S. A. Woodson, Folding mechanisms of group I ribozymes: Role of stability and contact order. Biochem. Soc. Trans. 30, 1166–1169 (2002). 12440997 10.1042/bst0301166

[18] J. L. Hougland, J. A. Piccirilli, M. Forconi, J. Lee, D. Herschlag, How the group I intron works: A case study of RNA structure and function, in *The RNA World,* R. F. Gesteland, J. F. Atkins, T. R. Cech, Ed. (Cold Spring Harbor Laboratory Press, 2006), pp. 133–205.

[19] P. P. Zarrinkar, J. R. Williamson, Kinetic intermediates in RNA folding. Science 265, 918–924 (1994). 8052848 10.1126/science.8052848

[20] W. D. Downs, T. R. Cech, Kinetic pathway for folding of the *Tetrahymena* ribozyme revealed by three UV-inducible crosslinks. RNA 2, 718–732 (1996). 8756414 PMC1369410

[21] V. L. Emerick, S. A. Woodson, Fingerprinting the folding of a group I precursor RNA. Proc. Natl. Acad. Sci. U.S.A. 91, 9675–9679 (1994). 7937871 10.1073/pnas.91.21.9675 PMC44879

[22] R. Russell, D. Herschlag, Probing the folding landscape of the *Tetrahymena* ribozyme: Commitment to form the native conformation is late in the folding pathway. J. Mol. Biol. 308, 839–851 (2001). 11352576 10.1006/jmbi.2001.4751

[23] R. Russell, R. Das, H. Suh, K. J. Travers, A. Laederach, M. A. Engelhardt, D. Herschlag, The paradoxical behavior of a highly structured misfolded intermediate in RNA folding. J. Mol. Biol. 363, 531–544 (2006). 16963081 10.1016/j.jmb.2006.08.024

[24] J. Pan, S. A. Woodson, Folding intermediates of a self-splicing RNA: Mispairing of the catalytic core. J. Mol. Biol. 280, 597–609 (1998). 9677291 10.1006/jmbi.1998.1901

[25] J. Pan, M. L. Deras, S. A. Woodson, Fast folding of a ribozyme by stabilizing core interactions: Evidence for multiple folding pathways in RNA. J. Mol. Biol. 296, 133–144 (2000). 10656822 10.1006/jmbi.1999.3439

[26] A. Punjani, J. L. Rubinstein, D. J. Fleet, M. A. Brubaker, cryoSPARC: Algorithms for rapid unsupervised cryo-EM structure determination. Nat. Methods 14, 290–296 (2017). 28165473 10.1038/nmeth.4169

[27] S. H. W. Scheres, RELION: Implementation of a Bayesian approach to cryo-EM structure determination. J. Struct. Biol. 180, 519–530 (2012). 23000701 10.1016/j.jsb.2012.09.006 PMC3690530

[28] A. Punjani, D. J. Fleet, 3D variability analysis: Resolving continuous flexibility and discrete heterogeneity from single particle cryo-EM. J. Struct. Biol. 213, 107702 (2021). 33582281 10.1016/j.jsb.2021.107702

[29] D. Liebschner, P. V. Afonine, M. L. Baker, G. Bunkóczi, V. B. Chen, T. I. Croll, B. Hintze, L. W. Hung, S. Jain, A. J. McCoy, N. W. Moriarty, R. D. Oeffner, B. K. Poon, M. G. Prisant, R. J. Read, J. S. Richardson, D. C. Richardson, M. D. Sammito, O. V. Sobolev, D. H. Stockwell, T. C. Terwilliger, A. G. Urzhumtsev, L. L. Videau, C. J. Williams, P. D. Adams, Macromolecular structure determination using x-rays, neutrons and electrons: Recent developments in Phenix. Acta Crystallogr. D Struct. Biol. 75, 861–877 (2019). 31588918 10.1107/S2059798319011471 PMC6778852

[30] P. Emsley, K. Cowtan, Coot: Model-building tools for molecular graphics. Acta Crystallogr. D Biol. Crystallogr. 60, 2126–2132 (2004). 15572765 10.1107/S0907444904019158

[31] D. Mitchell III, I. Jarmoskaite, N. Seval, S. Seifert, R. Russell, The long-range P3 helix of the Tetrahymena ribozyme is disrupted during folding between the native and misfolded conformations. J. Mol. Biol. 425, 2670–2686 (2013). 23702292 10.1016/j.jmb.2013.05.008 PMC3706569

[32] R. Russell, I. S. Millett, S. Doniach, D. Herschlag, Small angle x-ray scattering reveals a compact intermediate in RNA folding. Nat. Struct. Biol. 7, 367–370 (2000). 10802731 10.1038/75132

[33] D. Herschlag, S. Bonilla, N. Bisaria, The story of RNA folding, as told in epochs. Cold Spring Harb. Perspect. Biol. 10, (2018). 10.1101/cshperspect.a032433 PMC6169817 30275276

[34] J. Frank, Time-resolved cryo-electron microscopy: Recent progress. J. Struct. Biol. 200, 303–306 (2017). 28625887 10.1016/j.jsb.2017.06.005 PMC5732889

[35] E. F. Pettersen, T. D. Goddard, C. C. Huang, G. S. Couch, D. M. Greenblatt, E. C. Meng, T. E. Ferrin, UCSF Chimera--A visualization system for exploratory research and analysis. J. Comput. Chem. 25, 1605–1612 (2004). 15264254 10.1002/jcc.20084

[36] S. Lyskov, F. C. Chou, S. Ó. Conchúir, B. S. der, K. Drew, D. Kuroda, J. Xu, B. D. Weitzner, P. D. Renfrew, P. Sripakdeevong, B. Borgo, J. J. Havranek, B. Kuhlman, T. Kortemme, R. Bonneau, J. J. Gray, R. Das, Serverification of molecular modeling applications: The rosetta online server that includes everyone (ROSIE). PLOS ONE 8, e63906 (2013). 23717507 10.1371/journal.pone.0063906 PMC3661552

[37] E. F. Pettersen, T. D. Goddard, C. C. Huang, E. C. Meng, G. S. Couch, T. I. Croll, J. H. Morris, T. E. Ferrin, UCSF ChimeraX: Structure visualization for researchers, educators, and developers. Protein Sci. 30, 70–82 (2021). 32881101 10.1002/pro.3943 PMC7737788

[38] D. Svergun, C. Barberato, M. H. J. Koch, CRYSOL - A program to evaluate x-ray solution scattering of biological macromolecules from atomic coordinates. J. Appl. Cryst. 28, 768–773 (1995).

[39] R. T. Kidmose, J. Juhl, P. Nissen, T. Boesen, J. L. Karlsen, B. P. Pedersen, *Namdinator* - automatic molecular dynamics flexible fitting of structural models into cryo-EM and crystallography experimental maps. IUCrJ 6, 526–531 (2019). 10.1107/S2052252519007619 PMC6608625 31316797

[40] S. Chen, G. McMullan, A. R. Faruqi, G. N. Murshudov, J. M. Short, S. H. W. Scheres, R. Henderson, High-resolution noise substitution to measure overfitting and validate resolution in 3D structure determination by single particle electron cryomicroscopy. Ultramicroscopy 135, 24–35 (2013). 23872039 10.1016/j.ultramic.2013.06.004 PMC3834153

