# Supplementary material for: Cryo-EM reveals an entangled kinetic trap in the folding of a catalytic RNA
Source: Sci Adv. 2022 Aug 26;8(34):eabq4144. doi: 10.1126/sciadv.abq4144 (PMC9417180; doi:10.1126/sciadv.abq4144)
Supplement: Supplementary file 1 — Figs. S1 to S8 Table S1 References [file sciadv.abq4144_sm.pdf]

Science Advances
AAAS

Supplementary Materials for

Cryo-EM reveals an entangled kinetic trap in the folding of a catalytic RNA

Steve L. Bonilla *et al*.

Corresponding author: Email: Jeffrey S. Kieft, jeffrey.kieft@cuanschutz.edu

Sci. Adv. **8**, eabq4144 (2022)
DOI: 10.1126/sciadv.abq4144

The PDF file includes:

Figs. S1 to S8
Table S1
Legends for movies S1 and S2
References

Other Supplementary Material for this manuscript includes the following:

Movies S1 and S2

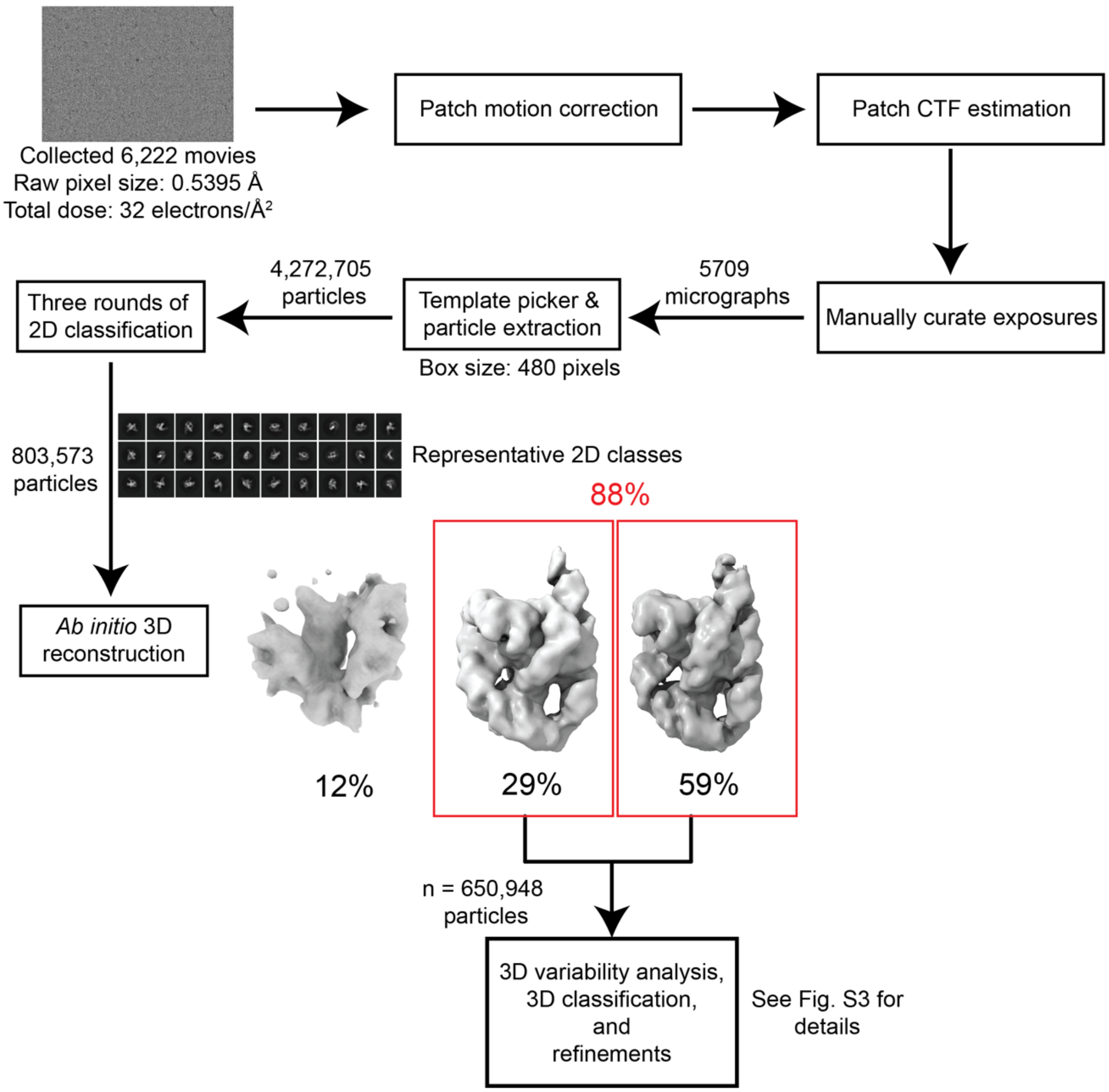

Fig. S1. Initial data analysis workflow. Data were collected with a 300 kV Thermo Scientific Krios TEM in super resolution mode and processed with cryoSPARC (*26*). Parameters are described in Materials and Methods. Three models were generated using *ab initio* reconstruction. Two of the volumes displayed structural features consistent with the TET ribozyme (red boxes). These volumes and their associated particles were subjected to 3D variability analysis, 3D classification, and refinements. See Fig. S3.

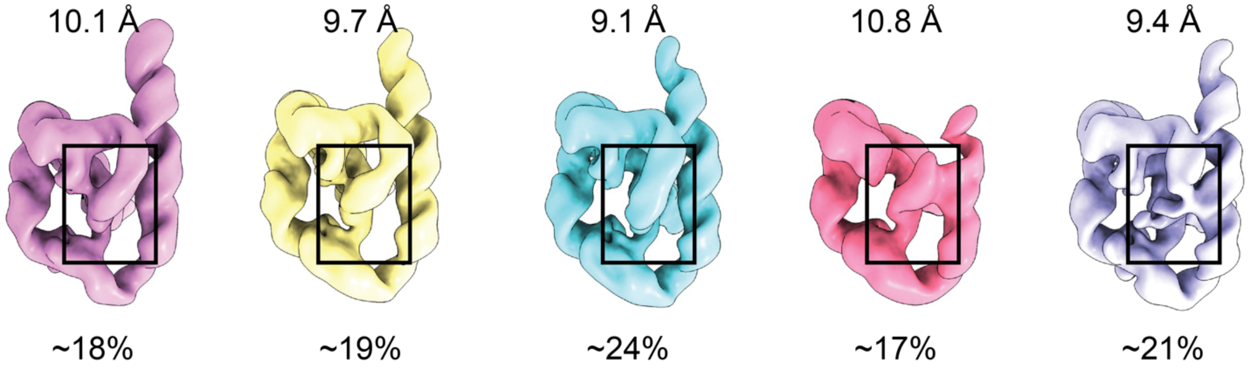

Fig. S2. Unsupervised 3D classification in Relion. Particles were classified into five classes. The resolution of each map is shown at the top and the fraction of particles at the bottom. The area corresponding to the catalytic core is boxed in each map.

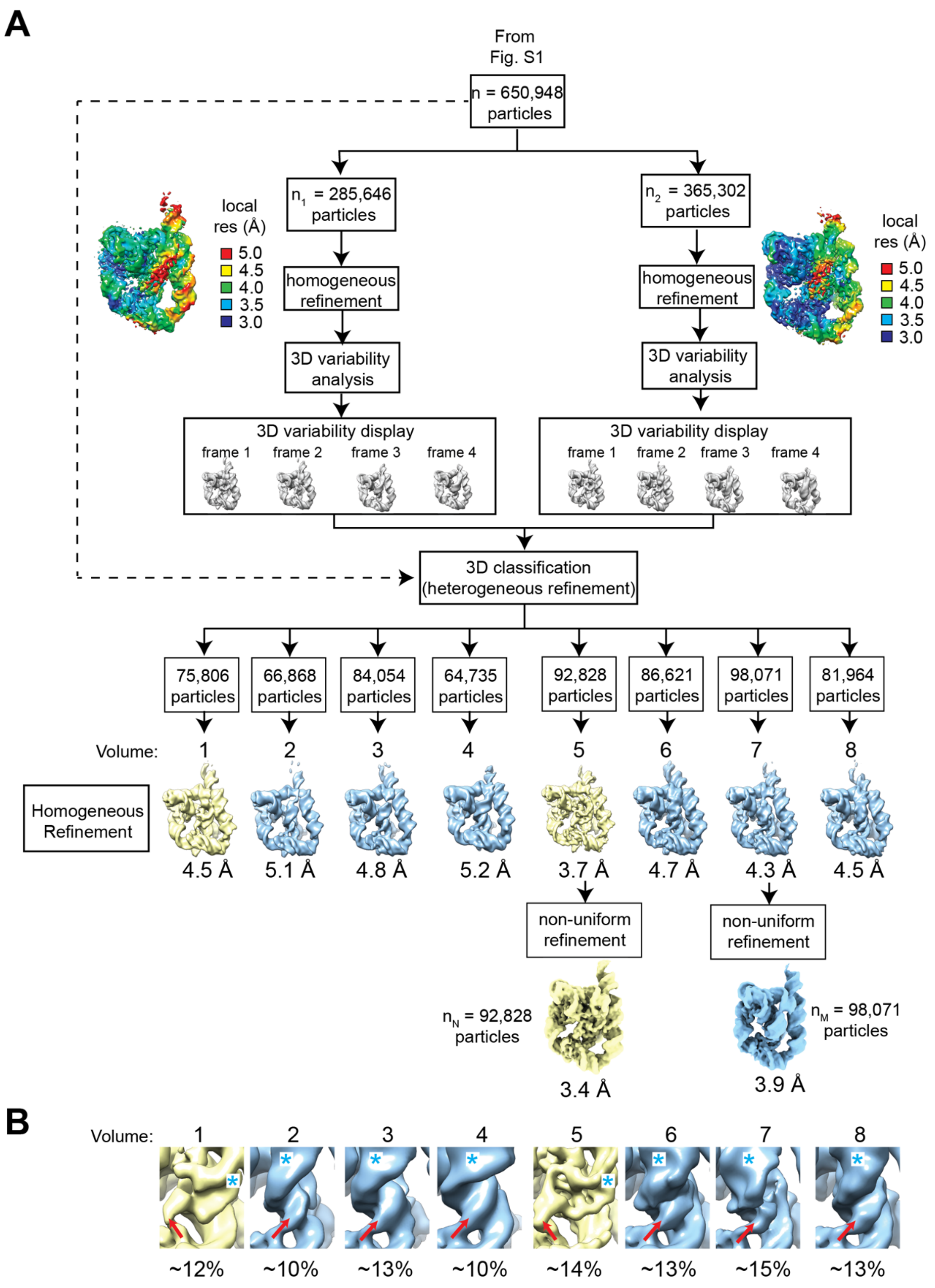

Fig. S3. Two ribozyme core conformations revealed by particle variability analysis. (A) cryoSPARC 3D variability analysis (*28*) was used to resolve conformational heterogeneity. Two *ab initio* models consistent with TET structure (Fig. S1) were refined and used as inputs for variability analyses. Four frames along the first principal component were generated for each input map. The total particles (n = 650,948) were re-classified across the eight frames using 'heterogeneous refinement' in cryoSPARC. (B) Close-up of the cores of refined maps generated variability analyses. Inspection of the maps revealed two conformational classes (yellow vs. blue maps). Major differences were observed in the direction of J8/7 density (red arrow) and orientation of the P7 minor groove (cyan asterisk). Further analysis revealed that the first class (yellow, ~26% of particles) corresponds to the N state and that the second class (blue, ~74% of particles) corresponds to the M state.

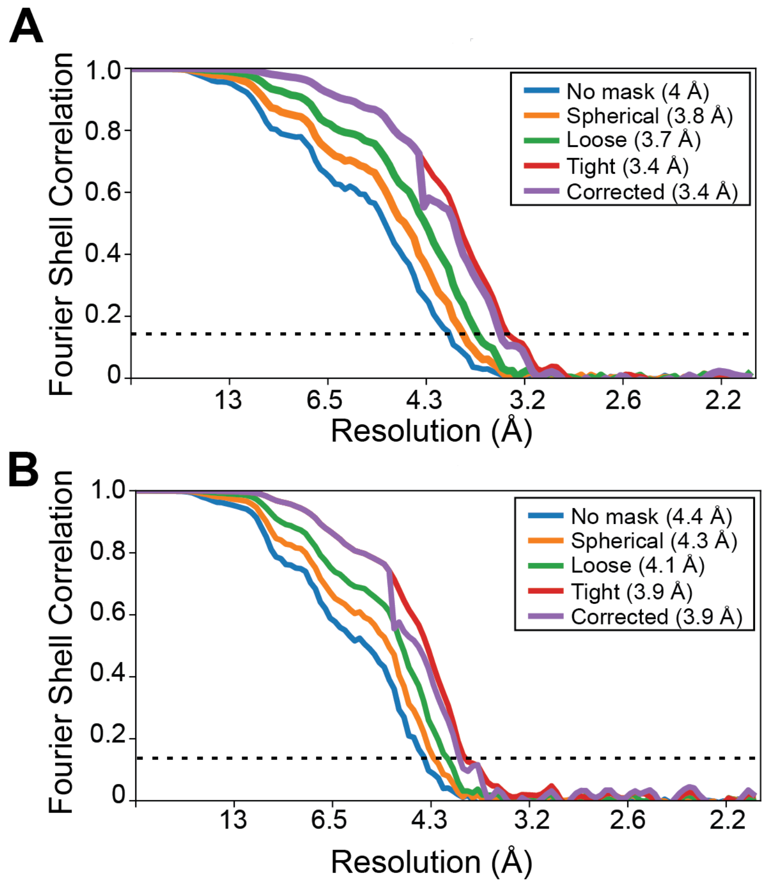

Fig. S4. Calculation of map resolutions. Fourier Shell Correlation (FSC) curves for the refinement of the N (A) and M (B) states of TET, generated by cryoSPARC (*26*). The resolutions reported were estimated using half maps and gold standard FSC (GSFSC) of 0.143 and corrected using high-resolution noise substitution (*40*) to measure the amount of noise overfitting.

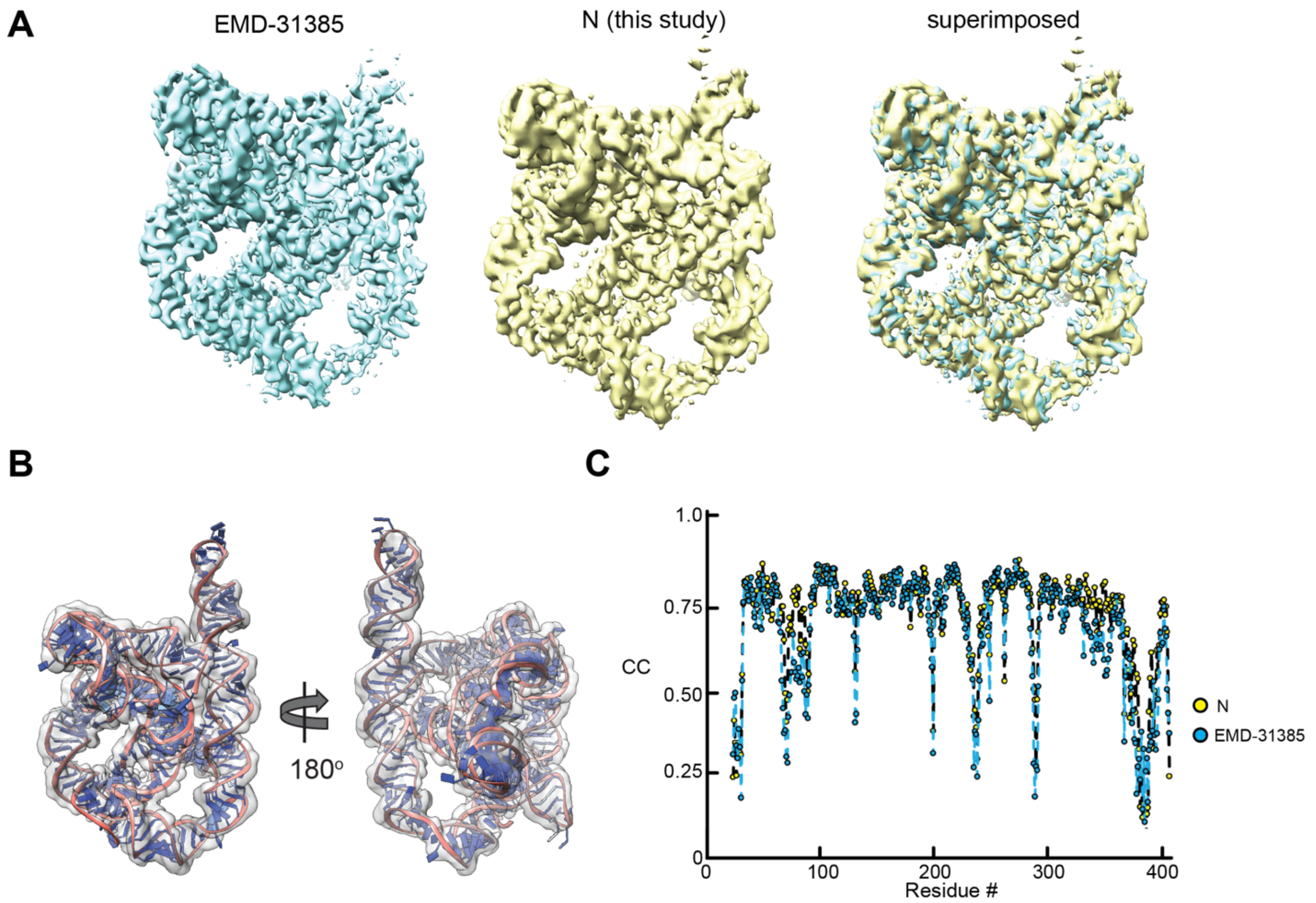

Fig. S5. Map of N agrees with published structure of *apo* L-21 ScaI TET ribozyme. (A) Comparison of one of the conformations revealed in this study (folded at 25°C) to one generated in previous studies (folded at 50°C) (EMD-31385) (*12*). The correlation between the maps is 0.94, as calculated with UCSF Chimera (*35*). (B) Published structure of TET in N state (PDB ID: 7ez0) docked into map of N generated in this study (CC_{mask} = 0.77). (C) Correlation per residue was similar between the published map of N (EMD-31385, blue) and the published structure (PDB ID: 7ez0) and the map of N generated in this study (yellow) and the published structure.

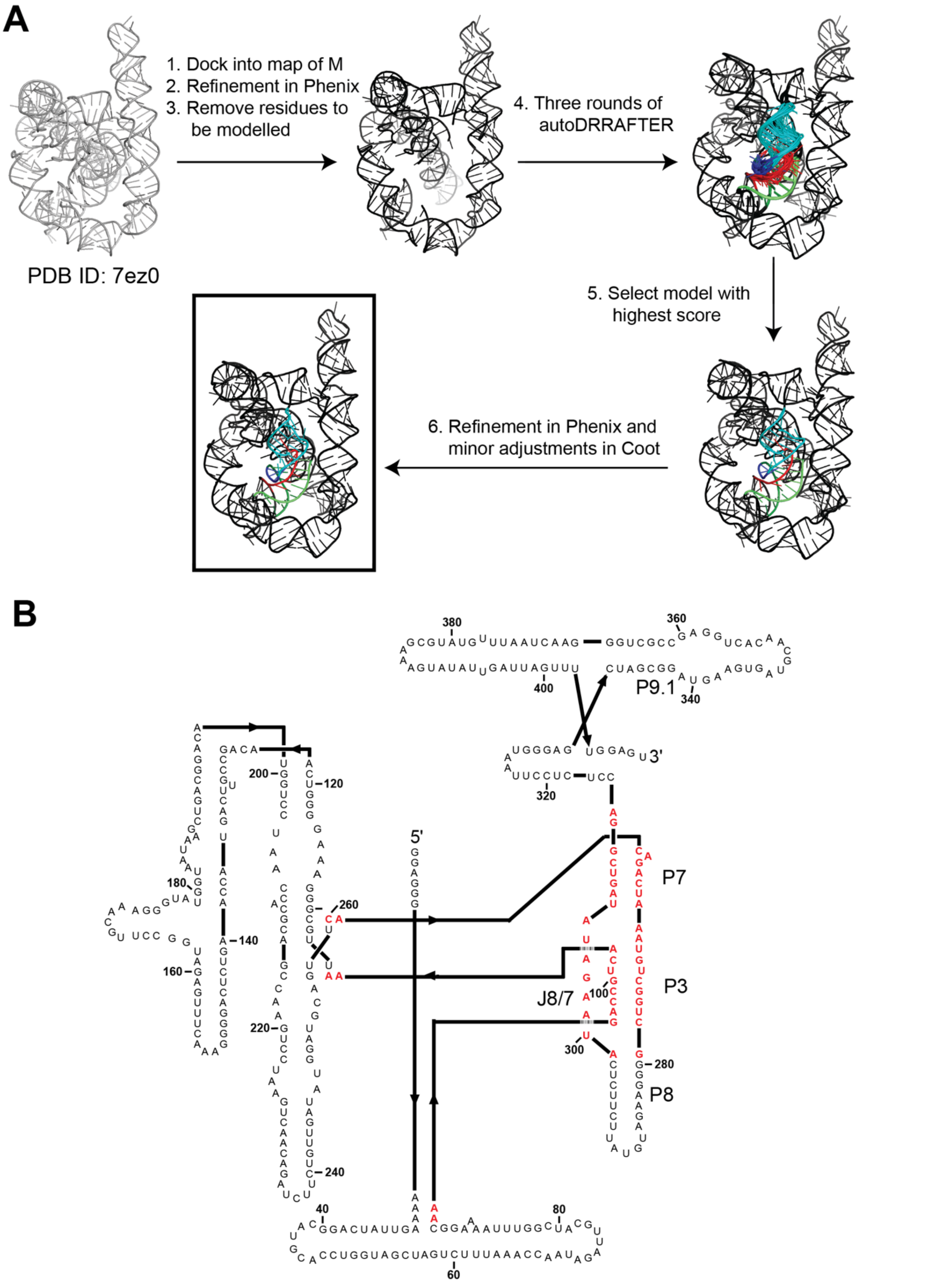

Fig. S6. Structural modelling of M. (A) Workflow followed to model the structure of M using autoDRRAFTER (*13*), Phenix (*29*), and Coot (*30*). (B) Regions of TET modeled. Nucleotides that were removed from published structure of TET (PDB ID: 7ez0) and were modeled de *novo* by fragment assembly in autoDRRAFTER are colored red.

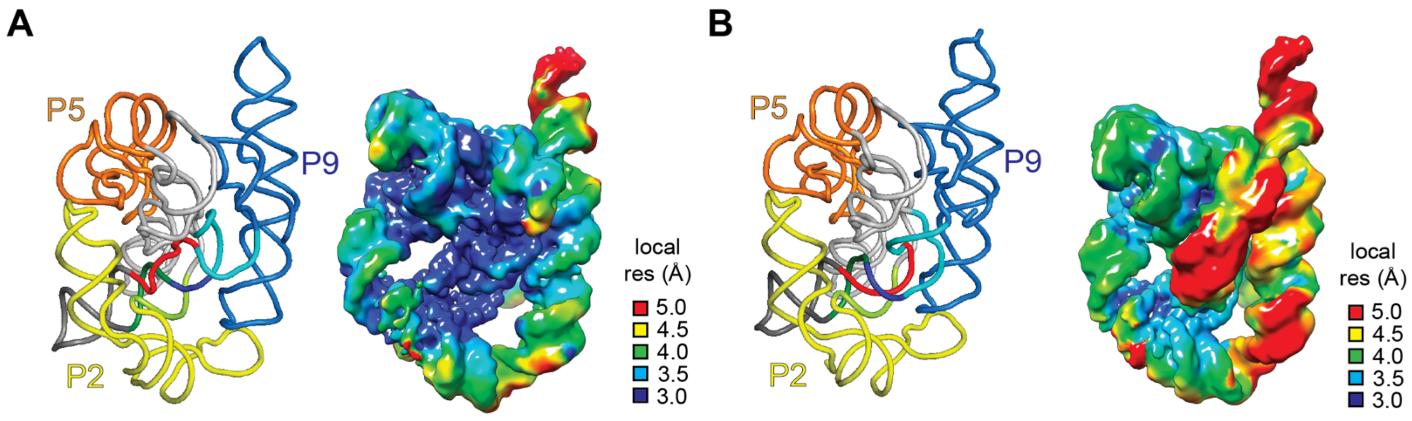

Fig. S7. Local resolution of N and M cryo-EM maps. (A) Structure (PDB ID: 7ez0) and cryo-EM map of N colored according to its local resolution. (B) Structure and cryo-EM map of M colored according to its local resolution. Threshold for local Fourier Shell Correlation (FSC) resolution was set to 0.143.

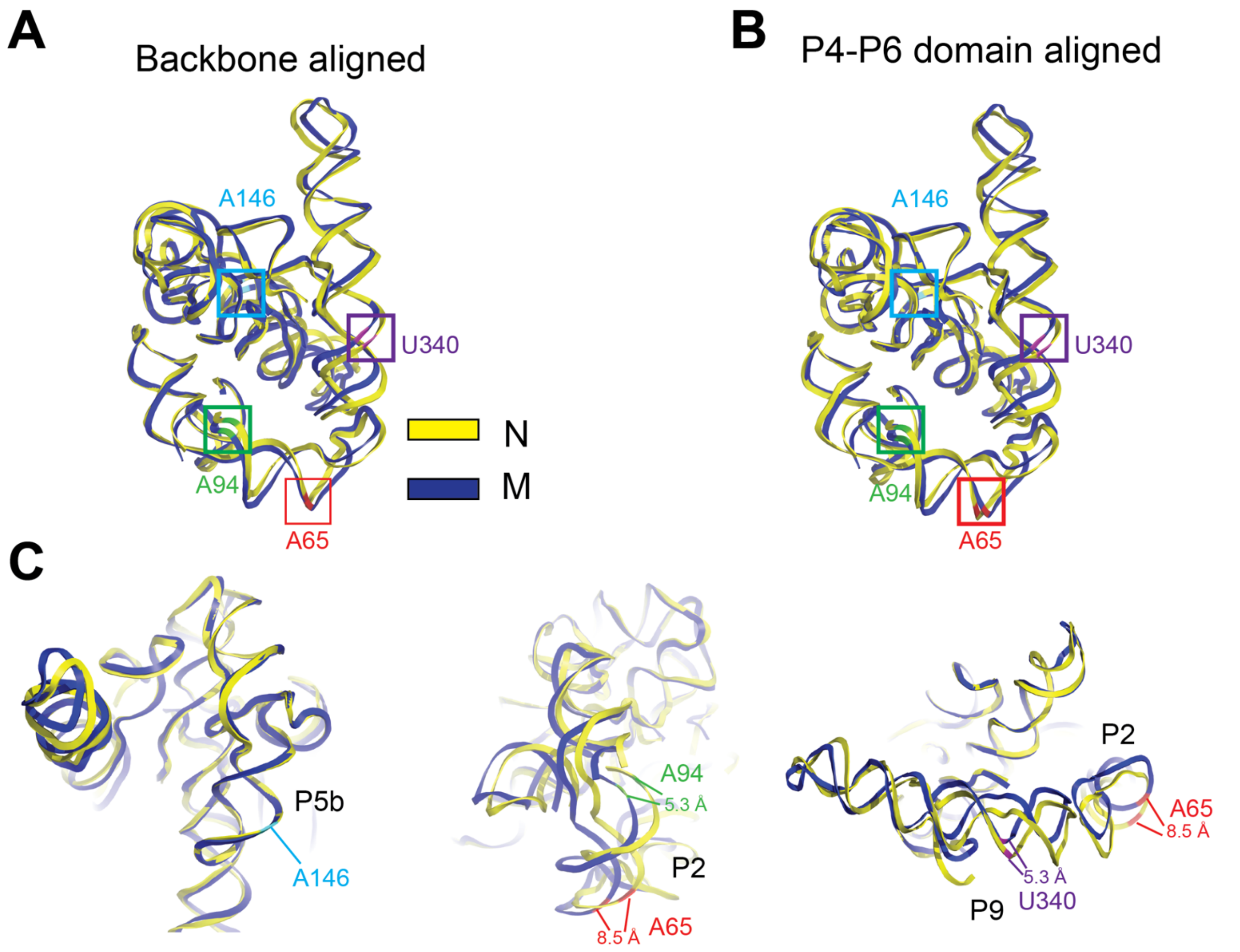

Fig. S8. Differences in the relative position of peripheral domains between N and M. (A) Superposition of the backbones of N and M ($RMSD_{backbone}$: 2.43 Å; 3758 atoms aligned). Regions that are distinct in the misfolded state were removed and did not contribute to the alignment. Nucleotides compared in (C) are colored and boxed. (B) Superposition of N and M based on the structural alignment of the P4-P6 domain (nts. 107 to 258; RMSD: 0.995Å). Colors are as in (A). (C) Superimposed structures (B) viewed from three angles. Distances measured between phosphates.

Table S1. Data collection parameters and model statistics.

	TET M State	TET N State
Microscope	Krios	Krios
Voltage (KeV)	300	300
Camera	Falcon 3 DED	Falcon 3 DED
Pixel size at detector (Å/pixel)	0.5395	0.5395
Total electron exposure (e-/Å^2)	32	32
No. of frames collected during exposure	46	46
Defocus range (µm)	0.8-2.0	0.8-2.0
Automation software	SerialEM	SerialEM
Tilt angle (°)	0	0
Energy filter slit width (eV)	20	20
Micrographs collected (no.)	6,222	6,222
Total extracted particles (no.)	4,272,705	4,272,702
**Reconstruction**		
Final refined particles (no.)	98,071	92,828
Point group	C1	C1
Resolution (Å) FSC: 0.143	3.9	3.4
Map sharpening *B* factor (Å^2)	167.1	121.9
Map sharpening methods	cryoSPARC global sharpening	cryoSPARC global sharpening
**Model Composition**		
Protein	0	-
RNA	386	-
**Model Refinement**		
Refinement package	Phenix	-
- Real or reciprocal space	Real	-
Model-Map scores		
- CC (box)	0.76	-
- CC (mask)	0.74	-
- CC (volume)	0.73	-
- CC (peaks)	0.66	-
R.m.s deviations from ideal values		
- Bond lengths (Å)	0.001	-
- Bond angles (°)	0.412	-
**Model Validation**		
MolProbity score	2.51	-
Clashscore	6.61	-

Movie Legends

Movie S1. Conformational heterogeneity localized to the core of the ribozyme. Particle 3D variability analysis in cryoSPARC was used to reveal heterogeneity at the core. Video shows four frames along the first principal component.

Movie S2. Conformational differences between M and N states. Video displays an example of the type of rearrangements necessary to transition from the N to the M states. Modelling was done by rearranging the structure manually.

REFERENCES AND NOTES

1. D. Herschlag, RNA chaperones and the RNA folding problem. *J. Biol. Chem.* **270**, 20871–20874 (1995).

2. D. Thirumalai, N. Lee, S. A. Woodson, D. Klimov, Early events in RNA folding. *Annu. Rev. Phys. Chem.* **52**, 751–762 (2001).

3. D. K. Treiber, J. R. Williamson, Exposing the kinetic traps in RNA folding. *Curr. Opin. Struct. Biol.* **9**, 339–345 (1999).

4. S. A. Woodson, Recent insights on RNA folding mechanisms from catalytic RNA. *Cell. Mol. Life Sci.* **57**, 796–808 (2000).

5. S. A. Walstrum, O. C. Uhlenbeck, The self-splicing RNA of *Tetrahymena* is trapped in a less active conformation by gel purification. *Biochemistry* **29**, 10573–10576 (1990).

6. T. Pan, T. R. Sosnick, Intermediates and kinetic traps in the folding of a large ribozyme revealed by circular dichroism and UV absorbance spectroscopies and catalytic activity. *Nat. Struct. Biol.* **4**, 931–938 (1997).

7. D. M. Chadalavada, S. M. Knudsen, S. Nakano, P. C. Bevilacqua, A role for upstream RNA structure in facilitating the catalytic fold of the genomic hepatitis delta virus ribozyme. *J. Mol. Biol.* **301**, 349–367 (2000).

8. R. Russell, RNA misfolding and the action of chaperones. *Front. Biosci.* **13**, 1–20 (2008).

9. S. A. Woodson, S. Panja, A. Santiago-Frangos, Proteins that chaperone RNA regulation. *Microbiol. Spectr.* **6**, 10.1128/microbiolspec.RWR-0026-2018 (2018).

10. M. L. Rodgers, S. A. Woodson, A roadmap for rRNA folding and assembly during transcription. *Trends Biochem. Sci.* **46**, 889–901 (2021).

11. S. L. Bonilla, M. E. Sherlock, A. MacFadden, J. S. Kieft, A viral RNA hijacks host machinery using dynamic conformational changes of a tRNA-like structure. *Science* **374**, 955–960 (2021).

12. Z. Su, K. Zhang, K. Kappel, S. Li, M. Z. Palo, G. D. Pintilie, R. Rangan, B. Luo, Y. Wei, R. Das, W. Chiu, Cryo-EM structures of full-length *Tetrahymena* ribozyme at 3.1 Å resolution. *Nature* **596**, 603–607 (2021).

13. K. Kappel, K. Zhang, Z. Su, A. M. Watkins, W. Kladwang, S. Li, G. Pintilie, V. V. Topkar, R. Rangan, I. N. Zheludev, J. D. Yesselman, W. Chiu, R. Das, Accelerated cryo-EM-guided determination of three-dimensional RNA-only structures. *Nat. Methods* **17**, 699–707 (2020).

14. D. Liu, F. A. Thélot, J. A. Piccirilli, M. Liao, P. Yin, Sub-3-Å cryo-EM structure of RNA enabled by engineered homomeric self-assembly. *Nat. Methods* **19**, 576–585 (2022).

15. K. Kruger, P. J. Grabowski, A. J. Zaug, J. Sands, D. E. Gottschling, T. R. Cech, Self-splicing RNA: Autoexcision and autocyclization of the ribosomal RNA intervening sequence of *Tetrahymena*. *Cell* **31**, 147–157 (1982).

16. T. R. Cech, Ribozymes, the first 20 years. *Biochem. Soc. Trans.* **30**, 1162–1166 (2002).

17. S. A. Woodson, Folding mechanisms of group I ribozymes: Role of stability and contact order. *Biochem. Soc. Trans.* **30**, 1166–1169 (2002).

18. J. L. Hougland, J. A. Piccirilli, M. Forconi, J. Lee, D. Herschlag, How the group I intron works: A case study of RNA structure and function, in *The RNA World,* R. F. Gesteland, J. F. Atkins, T. R. Cech, Ed. (Cold Spring Harbor Laboratory Press, 2006), pp. 133–205.

19. P. P. Zarrinkar, J. R. Williamson, Kinetic intermediates in RNA folding. *Science* **265**, 918–924 (1994).

20. W. D. Downs, T. R. Cech, Kinetic pathway for folding of the *Tetrahymena* ribozyme revealed by three UV-inducible crosslinks. *RNA* **2**, 718–732 (1996).

21. V. L. Emerick, S. A. Woodson, Fingerprinting the folding of a group I precursor RNA. *Proc. Natl. Acad. Sci. U.S.A.* **91**, 9675–9679 (1994).

22. R. Russell, D. Herschlag, Probing the folding landscape of the *Tetrahymena* ribozyme: Commitment to form the native conformation is late in the folding pathway. *J. Mol. Biol.* **308**, 839–851 (2001).

23. R. Russell, R. Das, H. Suh, K. J. Travers, A. Laederach, M. A. Engelhardt, D. Herschlag, The paradoxical behavior of a highly structured misfolded intermediate in RNA folding. *J. Mol. Biol.* **363**, 531–544 (2006).

24. J. Pan, S. A. Woodson, Folding intermediates of a self-splicing RNA: Mispairing of the catalytic core. *J. Mol. Biol.* **280**, 597–609 (1998).

25. J. Pan, M. L. Deras, S. A. Woodson, Fast folding of a ribozyme by stabilizing core interactions: Evidence for multiple folding pathways in RNA. *J. Mol. Biol.* **296**, 133–144 (2000).

26. A. Punjani, J. L. Rubinstein, D. J. Fleet, M. A. Brubaker, cryoSPARC: Algorithms for rapid unsupervised cryo-EM structure determination. *Nat. Methods* **14**, 290–296 (2017).

27. S. H. W. Scheres, RELION: Implementation of a Bayesian approach to cryo-EM structure determination. *J. Struct. Biol.* **180**, 519–530 (2012).

28. A. Punjani, D. J. Fleet, 3D variability analysis: Resolving continuous flexibility and discrete heterogeneity from single particle cryo-EM. *J. Struct. Biol.* **213**, 107702 (2021).

29. D. Liebschner, P. V. Afonine, M. L. Baker, G. Bunkóczi, V. B. Chen, T. I. Croll, B. Hintze, L. W. Hung, S. Jain, A. J. McCoy, N. W. Moriarty, R. D. Oeffner, B. K. Poon, M. G. Prisant, R. J. Read, J. S. Richardson, D. C. Richardson, M. D. Sammito, O. V. Sobolev, D. H. Stockwell, T. C. Terwilliger, A. G. Urzhumtsev, L. L. Videau, C. J. Williams, P. D. Adams, Macromolecular structure determination using x-rays, neutrons and electrons: Recent developments in Phenix. *Acta Crystallogr. D Struct. Biol.* **75**, 861–877 (2019).

30. P. Emsley, K. Cowtan, Coot: Model-building tools for molecular graphics. *Acta Crystallogr. D Biol. Crystallogr.* **60**, 2126–2132 (2004).

31. D. Mitchell III, I. Jarmoskaite, N. Seval, S. Seifert, R. Russell, The long-range P3 helix of the Tetrahymena ribozyme is disrupted during folding between the native and misfolded conformations. *J. Mol. Biol.* **425**, 2670–2686 (2013).

32. R. Russell, I. S. Millett, S. Doniach, D. Herschlag, Small angle x-ray scattering reveals a compact intermediate in RNA folding. *Nat. Struct. Biol.* **7**, 367–370 (2000).

33. D. Herschlag, S. Bonilla, N. Bisaria, The story of RNA folding, as told in epochs. *Cold Spring Harb. Perspect. Biol.* **10**, (2018).

34. J. Frank, Time-resolved cryo-electron microscopy: Recent progress. *J. Struct. Biol.* **200**, 303–306 (2017).

35. E. F. Pettersen, T. D. Goddard, C. C. Huang, G. S. Couch, D. M. Greenblatt, E. C. Meng, T. E. Ferrin, UCSF Chimera--A visualization system for exploratory research and analysis. *J. Comput. Chem.* **25**, 1605–1612 (2004).

36. S. Lyskov, F. C. Chou, S. Ó. Conchúir, B. S. der, K. Drew, D. Kuroda, J. Xu, B. D. Weitzner, P. D. Renfrew, P. Sripakdeevong, B. Borgo, J. J. Havranek, B. Kuhlman, T. Kortemme, R. Bonneau, J. J. Gray, R. Das, Serverification of molecular modeling applications: The rosetta online server that includes everyone (ROSIE). *PLOS ONE* **8**, e63906 (2013).

37. E. F. Pettersen, T. D. Goddard, C. C. Huang, E. C. Meng, G. S. Couch, T. I. Croll, J. H. Morris, T. E. Ferrin, UCSF ChimeraX: Structure visualization for researchers, educators, and developers. *Protein Sci.* **30**, 70–82 (2021).

38. D. Svergun, C. Barberato, M. H. J. Koch, CRYSOL - A program to evaluate x-ray solution scattering of biological macromolecules from atomic coordinates. *J. Appl. Cryst.* **28**, 768–773 (1995).

39. R. T. Kidmose, J. Juhl, P. Nissen, T. Boesen, J. L. Karlsen, B. P. Pedersen, *Namdinator* - automatic molecular dynamics flexible fitting of structural models into cryo-EM and crystallography experimental maps. *IUCrJ* **6**, 526–531 (2019).

40. S. Chen, G. McMullan, A. R. Faruqi, G. N. Murshudov, J. M. Short, S. H. W. Scheres, R. Henderson, High-resolution noise substitution to measure overfitting and validate resolution in 3D structure determination by single particle electron cryomicroscopy. *Ultramicroscopy* **135**, 24–35 (2013).
